# Arterial Klotho Expression and FGF23 Effects on Vascular Calcification and Function

**DOI:** 10.1371/journal.pone.0060658

**Published:** 2013-04-05

**Authors:** Karolina Lindberg, Hannes Olauson, Risul Amin, Arvind Ponnusamy, Regina Goetz, Rebecca F. Taylor, Moosa Mohammadi, Ann Canfield, Karolina Kublickiene, Tobias E. Larsson

**Affiliations:** 1 Division of Renal Medicine, Department of Clinical Science, Intervention and Technology, Karolinska Institutet, Stockholm, Sweden; 2 Wellcome Trust Centre for Cell-Matrix Research, Institute of Cardiovascular Sciences, Faculty of Medical and Human Sciences, University of Manchester, Manchester, United Kingdom; 3 Department of Biochemistry and Molecular Pharmacology, New York University School of Medicine, New York, New York, United States of America; 4 Division of Obstetrics and Gynecology, Department of Clinical Science, Intervention and Technology, Karolinska Institutet, Stockholm, Sweden; 5 Department of Nephrology, Karolinska University Hospital, Stockholm, Sweden; National Cancer Institute, United States of America

## Abstract

Recent studies support a role for FGF23 and its co-receptor Klotho in cardiovascular pathology, yet the underlying mechanisms remain largely elusive. Herein, we analyzed the expression of Klotho in mouse arteries and generated a novel mouse model harboring a vascular smooth muscle cell specific deletion of Klotho (*Sm22-KL^−/−^*). Arterial Klotho expression was detected at very low levels with quantitative real-time PCR; Klotho protein levels were undetectable by immunohistochemistry and Western blot. There was no difference in arterial Klotho between *Sm22-KL^−/−^* and wild-type mice, as well as no changes in serum markers of mineral metabolism. Intravenous delivery of FGF23 elicited a rise in renal (0.005; p<0.01) but not arterial Egr-1 expression, a marker of Klotho-dependent FGF23 signaling. Further, the impact of FGF23 on vascular calcification and endothelial response was evaluated in bovine vascular smooth muscle cells (bVSMC) and in a murine *ex vivo* model of endothelial function, respectively. FGF23 treatment (0.125–2 ng/mL) did not modify calcification in bVSMCs or dilatory, contractile and structural properties in mice arterial specimen *ex vivo*. Collectively, these results demonstrate that FGF23-Klotho signaling is absent in mouse arteries and that the vascular response was unaffected by FGF23 treatment. Thus, our data do not support Klotho-mediated FGF23 effects in the vasculature although confirmative studies in humans are warranted.

## Introduction

The cardiovascular disease burden in patients with chronic kidney disease (CKD) is substantial and derives from numerous aggregating cardiovascular risk factors [1]. Recent clinical and observational data suggest that FGF23, a bone-derived hormone that regulates mineral metabolism, is linked to cardiovascular mortality as well as subclinical indices of cardiovascular pathology such as left ventricular hypertrophy, vascular calcification and endothelial dysfunction [2], [3], [4], [5], [6], [7].

Classical FGF23 endocrine signaling in the kidney entails binding and activation of an FGF-receptor in combination with the obligate co-receptor alpha-Klotho (Klotho) [8]. Disruption of FGF23-Klotho signaling is an early hallmark of CKD involving reduced Klotho tissue level and a reciprocal rise in circulating FGF23 [9]. It is currently not established whether the rise in FGF23 precedes the loss of Klotho or *vice versa*. However, the initial onset and gradually accelerating perturbation in FGF23-Klotho expression appear intimately connected with development of cardiovascular disease (CVD).

A link between FGF23-Klotho and CVD has recently been confirmed in experimental studies. As such, FGF23 was reported to exert Klotho-independent effects in the myocardium and directly contribute to left ventricular hypertrophy, a prevalent manifestation and well-established cardiovascular risk factor in CKD [10]. Furthermore, circulating Klotho derived from shedding of membrane-bound Klotho was shown to inhibit vascular calcification by preventing precipitation of calcium and phosphate in vascular smooth muscle cells [11]. Another recent study suggested that Klotho is locally expressed in vascular smooth muscle cells of human arteries, and protects against vascular calcification by mediating FGF23 inhibitory effects on matrix mineralization [12]. However, significant controversies prevail regarding the presence of membrane-bound Klotho in the vascular system and whether or not vascular tissue is directly responsive to FGF23 endocrine action.

To better define the role of FGF23-Klotho in cardiovascular pathology, we evaluated arterial Klotho expression in wild-type mice and in a novel mouse strain with a targeted deletion of Klotho in vascular smooth muscle cells (*Sm22-KL^−/−^*). Further, we tested whether FGF23 has direct short and long term effects on endothelial and vascular smooth muscle function *ex vivo* in mouse arteries and vascular calcification *in vitro* in bovine vascular smooth muscle cells (bVSMC).

## Materials and Methods

### Ethics Statement

All experiments were conducted in compliance with the guidelines of animal experiments at Karolinska Institutet and approved by Stockholms South Animal Experimental Ethical Comittee (Permit Number: 68∶10).

### Generation of Sm22-KL^−/−^ and β-KL^−/−^ mice

Mice with Klotho deletion in vascular smooth muscle cells were generated using Cre-Lox recombination as previously described [14]. Briefly, loxP sequences were introduced in the flanking regions of exon 2 of the Klotho gene resulting in a non-functioning Klotho protein in tissues expressing Cre recombinase. Male chimeras were generated and subsequently bred with wild-type females to generate Klotho-LoxP heterozygotes (Klotho^flox/+^). Klotho^flox/+^ were crossed with mice expressing Cre recombinase under the smooth muscle protein 22-alpha promoter (Tg(Tagln-cre)1Her/J; Jackson laboratory, ME, US). Klotho-LoxP heterozygous mice expressing Cre recombinase (Klotho^flox/+^, Sm22-Cre) were crossed with Klotho^flox/+^ to generate homozygous vascular-specific Klotho *null* mice (Klotho^flox/flox^, Sm22-Cre; *Sm22-KL* ^−/−^). Homozygous mice not expressing Cre (Klotho^flox/flox^) served as wild-type controls. To minimize the intra-litter variability, mice were intercrossed, making 50% of the offspring vascular-specific Klotho *null* mice and 50% wild-type mice, which were subsequently analyzed for their respective genotype. Mice with a systemic Klotho deletion (*β-KL^−/−^)* were generated using mice expressing Cre under the human beta-actin promotor (FVB/N-Tg(ACTB-cre)2Mrt/J; Jackson laboratory, ME, US) as previously described [14].

### Genotyping

Total DNA was extracted from tail biopsies using DirectPCR Lysis Reagent (Viagen, Los Angeles, CA). PCR amplification was carried out on a 2720 Thermal Cycler (Applied Biosystems, Carlsbad, CA) using HotStarTaq DNA Polymerase (Qiagen, Venlo, Netherlands). The PCR products were visualized on a 1% agarose gel with GelRed Nucleic Acid Gel Stain (Biotium, Hayward, CA). Sequences of the primers used for genotyping are listed in Table S1.

### Serum biochemistries

Serum calcium, phosphate and creatinine were measured using quantitative colorimetric assay kits (BioAssay/BioChain, Hayward, CA, US). Serum PTH was measured using a Mouse 1–84 PTH ELISA kit and FGF23 was measured using a Mouse FGF23 (c-term) ELISA kit (Immunotopics, San Clemente, CA, US). Serum 1,25-dihydroxy vitamin D was measured using EIA kit (Immunodiagnostic systems, Scottsdale, AZ, US).

### Analysis of Klotho expression in mouse arteries, DCT209 cells and bovine VSMCs

Mouse arteries and lungs were immediately after removal put in RNA-later (Qiagen, Venlo, Netherlands) and subsequently microdissected. Mouse distal convoluted tubule 209 cells (DCT209 – a kind gift from Dr. Frank Thévenod) were grown in 6-well plates in DMEM/Ham-F12 (Invitrogen) medium supplemented 5% FBS (Invitrogen), 50 µg/mL penicillin-streptomycin (Invitrogen) and 100 µg/100 mL neomycin (Sigma). Bovine VSMCs were maintained in 10% FCS-DMEM and grown until confluence, whereafter cells were treated +/−5 mM β-glycerophosphate (BGP) for up to 7 days. The arteries, and cell pellets of VSMCs and DCT209 cells were then homogenized using a TissueLyser LT (Qiagen) and RNA was extracted with E.Z.N.A. Total RNA Kit I (Omega Bio-tek, Norcross, GA). First strand cDNA synthesis was carried out using iScript cDNA Synthesis Kit (Bio-Rad, Hercules, CA). For quantitative real time PCR analysis the CFX96 Real-Time PCR Detection System and iQ SYBR Green Supermix (Bio-Rad) were used. The relative gene expression was calculated using the 2^−ΔΔ^ Cq method normalizing the gene of interest to β-actin in the same sample. Data is presented as relative fold-change compared to WT mice. Sequences of the primers used for quantitative real-time PCR are shown in Table S1.

For protein detection, aortas, femoral arteries, mesenteric arteries and pulmonary arteries from wild-type and *Sm22-KL* ^−/−^ mice were dissected, fixed in 4% paraformaldehyde overnight and subsequently embedded in paraffin. Kidneys from wild-type mice were used as positive controls. Four µm sections were deparaffinized, rehydrated and incubated with low pH antigen unmasking solution (Vector Laboratories, Peterborough, UK) in a 2100 retriever (PickCell laboratories). The sections were then immersed in 3% H_2_O_2_ in methanol, treated with 4% normal serum and blocked with Avidin and Biotin (Vector Laboratories). Sections were incubated with primary antibodies at 4°C overnight, followed by biotinylated secondary antibodies for 60 min and finally incubated with Vector ABC Reagent and developed with DAB substrate (Vector Laboratories). All slides were counterstained with Mayer's hematoxylin. The antibodies used were rat monoclonal anti-Klotho (KM2076 and KM2119, TransGenic Inc. Japan).

Western blotting of pooled aortas derived from wild-type or *Sm22-KL^−/−^* mice were performed as follows: aortas were homogenized in PBS-TDS buffer (PBS with 1% Triton X-100, 0.5% sodium deoxycholate, 0.1% SDS, 1 mM EDTA, 1 mM PMSF) using a Tissue Lyzer LT (Qiagen). Extracts were incubated on ice for 30 minutes, and centrifuged at 10 000 rpm for 10 min at 4°C. Supernatants were collected for further analysis. Protein quantification was carried out using BCA protein assay kit (Thermo Scientific, Rockford, IL, USA). Eight µg of protein was separated on an Any kD SDS-PAGE and electrotransferred to a Nitrocellulose membrane (iBlot Gel Transfer Stacks Nitrocellulos, Invitrogen). After blocking with ODYSSEY blocking buffer (Li-COR Bioscience, Lincoln, NE) membranes were sequentially incubated with primary and secondary antibodies. Primary antibodies were anti-Klotho (KM2076 and KM2119, TransGenic Inc.) and anti-β-actin (Sigma-Aldrich). The secondary antibodies were IRDye 680 Goat anti-Rat and IRdye 680 Goat anti-mouse (LI-COR Biosciences). Visualization was carried out using ODYSSEY Infrared imaging System (LI-COR Biosciences).

### Egr-1 expression in response to FGF23 injection

Recombinant FGF23 protein was produced as described elsewhere [22] and injected intravenously into the tail vein (0.15 mg/kg). 0.9% NaCl was used as negative control. Mice were sacrificed 30 minutes after injection and aortas and kidneys were immediately dissected and snap frozen in liquid nitrogen. Analysis of Egr-1 was carried out using real-time qPCR as described above.

### *In vitro* assay of vascular calcification

Bovine VSMCs (passage 10), were plated at 1×10^4^ cells/cm^2^ in 6-well plates and maintained in 10% FCS-DMEM until 90% confluence, whereafter they were cultured in 10% FCS-DMEM containing 5 mM β-glycerophosphate +/− FGF23 (0.125–2 ng/mL). Medium was changed every three days and cells were stained for mineralization with Alizarin red after 8, 9 and 10 days. Mineralization was quantified by eluting the dye and measuring the absorbance. All experiments were performed in triplicates.

### *Ex vivo* testing of vascular response to FGF23

Contraction and relaxation responses to vasoactive drugs (phenylephrine (PHE), acetylcholine (ACh), thromboxane A2 (TXA_2_) analog U46619 and sodium nitroprusside (SNP)) were investigated in small mesenteric arteries of wild-type mice *ex vivo* after short and prolonged incubation with FGF23 or vehicle. Passive length-tension relations were assessed after prolonged incubation. The full protocol description is provided in Material and Methods S1.

### Statistical Analysis

GraphPad Prism 5.0 (GraphPad Software Inc, CA, US) was used for statistical analysis. Gaussian distribution was tested using D'Agostino and Pearson omnibus normality test. Variables fulfilling the criteria for normal distribution were tested with two-tailed t-test. Non-normally distributed variables were compared using Mann-Whitney test. P-values<0.05 were considered statistically significant.

## Results

### Generation of Sm22-KL^−/−^ and β-KL^−/−^ mice

**Figure 1:**
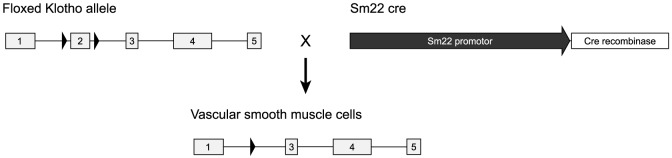
Targeted deletion of Klotho in *Sm22-KL^**−/−**^* mice. Mice with loxP sites inserted into intron 1 and 2 of Klotho, were crossed with transgenic mice expressing Cre recombinase under the control of the mouse smooth muscle protein 22-alpha (Sm22) promoter, resulting in targeted deletion of the Klotho gene specifically in vascular smooth muscle cells by Cre recombination.

Mice with a tissue-specific deletion of Klotho in vascular smooth muscle cells (*Sm22-KL^−/−^*) and with a systemic Klotho deletion (*β-KL^−/−^*) were generated using Cre-LoxP recombination as described in Methods (Figure 1). Floxed Klotho mice were crossed with mice expressing Cre recombinase driven by the Sm22 promoter, which was previously shown to have Cre activity specifically in the vascular smooth muscle cells [13], or human β-actin promoter respectively. Expression of Cre recombinase in arteries of *Sm22-KL^−/−^* mice was confirmed using quantitative real-time PCR with specific primers against Cre (data not shown). Confirmation of non-functional Klotho alleles in the presence of Cre was demonstrated previously [14].

### Gross phenotype of adult Sm22-KL^−/−^ mice

Mice were analyzed at 8 weeks of age. *Sm22-KL^−/−^* mice were viable, fertile with normal gross phenotype and no apparent changes in mineral metabolism (Table 1) indicating that the vasculature is not a major target tissue for FGF23.

**Table 1:** Serum biochemistries in *Sm22-KL* ^−/−^ and wild-type mice.

Serum Biochemistries			
Parameters	WT (n = 10)	*Sm22-KL^**−/−**^* (n = 7)	*P* value
Calcium (mg/dL)	9.72 (9.09–10.53)	9.30 (8.07–10.43)	0.14
Creatinine (mg/dL)	0.45 (0.30–0.60)	0.50 (0.30–0.60)	0.58
Phosphate (mg/dL)	3.34 (2.96–3.77)	3.19 (2.91–3.39)	0.14
FGF23 (pg/mL)	127.6 (68.1–230.0)	115.7 (56.2–170.5)	0.91
PTH (pg/mL)	39.2 (27.5–86.8)	37.2 (24.8–98.1)	0.35

At 8 weeks of age *Sm22-KL^−/−^* mice had normal serum biochemistries reflecting mineral metabolism as compared to wild-type controls. Results are displayed as median (range).

### Klotho expression in mouse arteries

**Figure 2:**
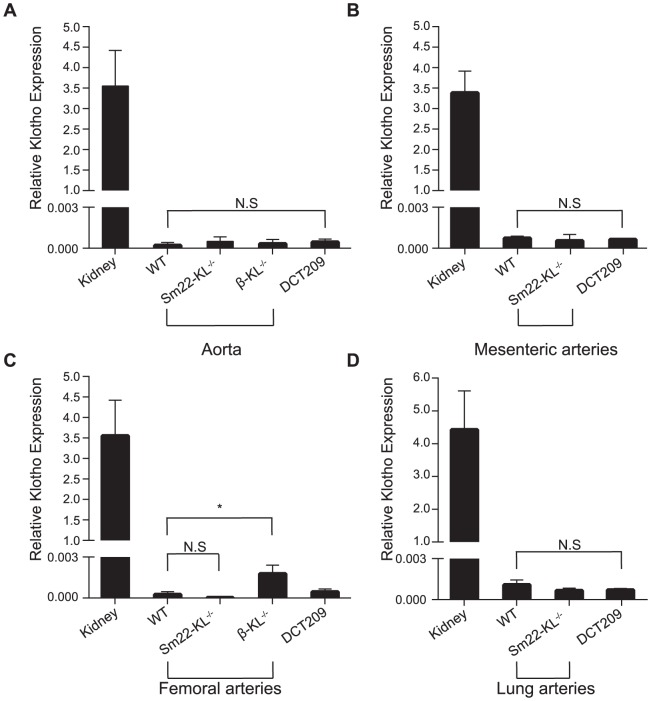
Klotho gene expression in mouse arteries. Gene expression of Klotho in kidney extracts, arteries and lung of wild-type mice, *Sm22-KL^−/−^* mice, *β-KL^−/−^* mice, and DCT209 cells. The Klotho transcript levels remained unchanged between wild-type and *Sm22-KL^**−/−**^* mice in all arteries investigated and were 7000 times lower when compared to kidney transcript levels. *Sm22-KL^−/−^* transcript levels remained at same levels or lower when compared with the *β-KL^−/−^* mice and the DCT209 cell line.

Aortas, femoral arteries, mesenteric arteries and lungs from wild-type (WT) and *Sm22-KL* ^−/−^ mice were dissected and prepared as described in Materials and Methods. In addition, aortas and femoral arteries were dissected from *β-KL* ^−/−^ mice to be used as negative controls. Klotho transcripts were detected at low levels by quantitative real-time PCR, however there was no difference in transcript levels between *Sm22-KL^−/−^* and wild-type mice (Figure 2A–D). In contrast, Klotho transcripts were higher in femoral arteries from *β-KL* ^−/−^ compared to WT mice (Figure 2C), whereas no difference was seen in aortas (Figure 2A). Arterial Klotho transcript levels were comparable to the expression in DCT 209 cells, and about 7000 fold lower than in kidneys (Figure 2).

**Figure 3:**
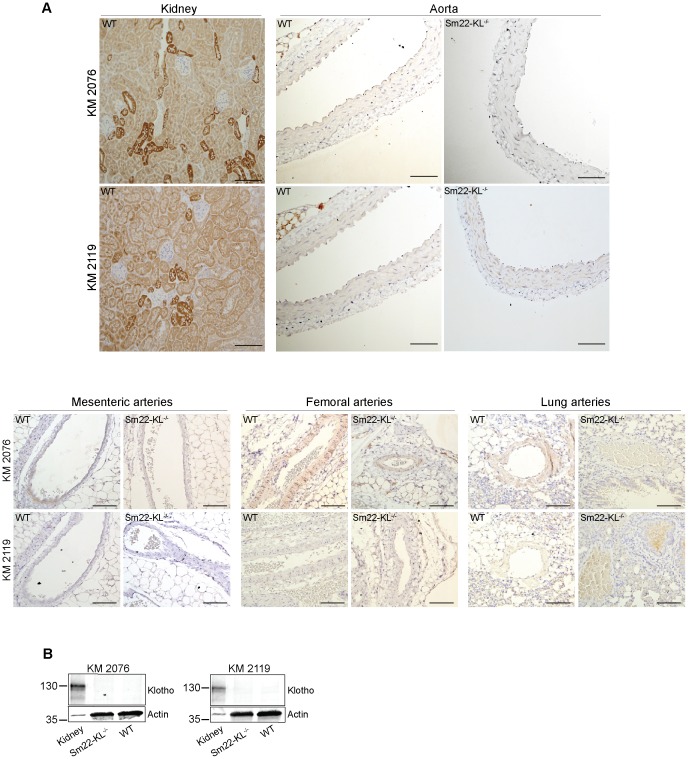
Immunohistochemical staining and Western blotting for expression of Klotho in different arteries from *Sm22-KL^−/−^* mice. (A) Klotho protein was undetectable in the vascular wall of different arteries (aorta, mesenteriac, femoral and lung arteries), as determined by immunohistochemical staining, when compared with a distinct positive staining for Klotho in kidney distal tubule. Upper panels show Klotho staining of aorta with two different antibodies (KM2076 and KM2119). Lower panel shows Klotho staining of mesenterial, femoral and lung arteries, using the same antibodies. All results are shown in 40x magnification. Scale bar, 10 µM. (**B**) Immunoblot analysis of pooled whole aortic lysates from three wild-type and three *Sm22-KL^−/−^* mice revealed no Klotho protein (using two different antibodies; KM2076 and KM2119), when compared with the positive control of whole kidney extracts from wild-type mice, which showed a strong band for Klotho at 130 kDa.

Immunostaining revealed no detectable Klotho in the arterial wall from WT or *Sm22-KL^−/−^* mice, using two different primary antibodies, whereas a strong signal was found in the kidney distal tubules (Figure 3A). Western blotting for Klotho similarly did not provide evidence for protein expression in aortas from either genotype (Figure 3B).

### Egr-1 response to FGF23 injection

**Figure 4:**
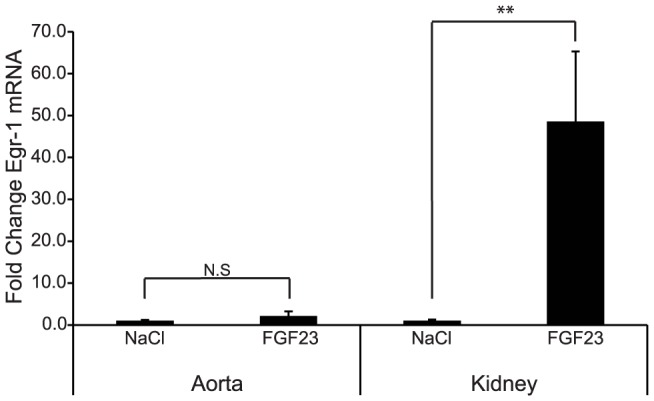
Vascular Egr-1 response to FGF23 injection. Transcript levels of Egr-1 were analyzed in aorta and kidneys from wild-type mice injected with 0.9% NaCl (n = 3) or 0.15 mg/kg FGF23 (n = 4). In contrast to the kidney, no distinct rise in Egr-1 mRNA was seen in aorta 30 min after an FGF23 injection. Data are presented as fold change, with NaCl set to 1.

To test whether mouse arteries expressed functional Klotho protein on the basis of low but detectable Klotho transcript level, WT mice were injected intravenously with a single dose of either FGF23 or saline, and the aorta and kidneys collected 30 after minutes. In contrast to kidneys, which demonstrated a distinct rise in Egr-1 mRNA, no increase in Egr-1 was found in the aortas from mice injected with FGF23 compared to saline (Figure 4).

### Impact of FGF23 on vascular calcification

**Figure 5:**
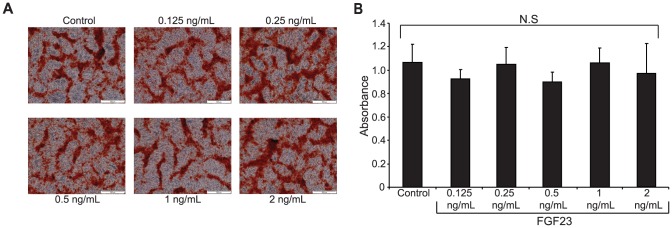
No effect of FGF23 on βGP-induced mineral deposition by bVSMCs. (**A**) bVSMCs were cultured in 6-well plates in 10% FCS-DMEM till 90% confluent, and then incubated in medium containing 5 mM βGP and mock transfected media (control), or with medium containing βGP supplemented with FGF23 in concentrations ranging from 0.125–2 ng/mL. Representative phase contrast images of cells stained with alizarin red on days 10 are shown. The red stain indicates areas of mineralization. Scale bar = 500 µm. (**B**) Alizarin red dye elution was performed in order to quantify mineralization. Results represent data from day 10. Data are shown as mean ± SEM (n = 3 individual samples).

Using an established *in vitro* assay of isolated bovine vascular smooth muscle cells (bVSMCs) [15], [16], [17], vascular calcification by FGF23 was investigated. Klotho mRNA level in these cells was very low but detectable, and remained unchanged by the presence of calcification medium for up to 7 days (Figure S1). FGF23 treatment (0.125–2 ng/mL) did not affect vascular calcification induced by addition of calcification medium to the cells during a time period of up to ten days (Figure 5). Similar negative results were seen at eight and nine days (data not shown).

### Impact of FGF23 on vascular function and structure

**Figure 6:**
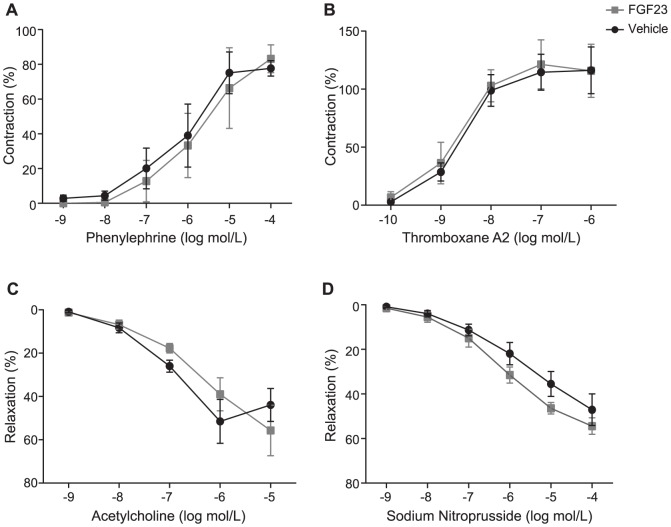
Short-term effects of FGF23 on induced contractions or relaxations. Short-term effects of FGF23 (6 ng/mL) or vehicle (DMSO 0.17% v/v) on phenylephrine (A), thromboxane A2 (TXA_2_) analog U46619 (B), acetylcholine (C) and sodium nitroprusside (D) induced contractions or relaxations, respectively. Data are shown as means ± SEM. Solid black lines indicate responses in the presence of vehicle and gray lines indicate responses in the presence of FGF23.

**Figure 7:**
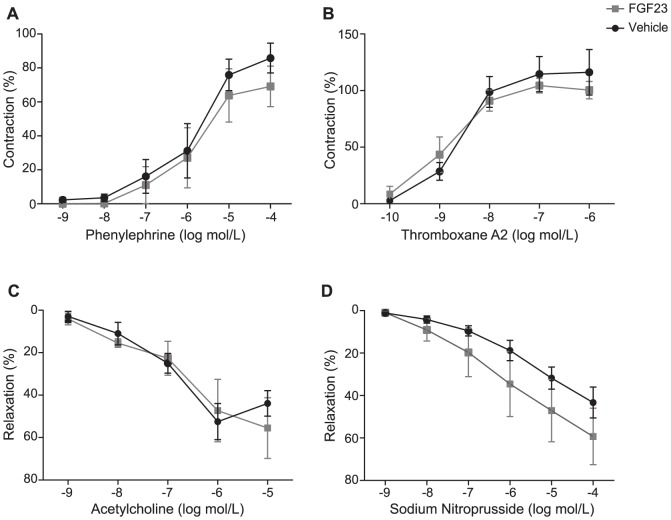
Long term effects of FGF23 on induced contractions or relaxations. Long term effects of FGF23 or vehicle (DMSO 0.17% v/v) on phenylephrine (A), thromboxane A2 (TXA_2_) analog U46619 (B), acetylcholine (C) and sodium nitroprusside (D) induced contractions or relaxations, respectively. Data are shown as means ± SEM. Solid black lines indicate responses in the presence of vehicle and gray lines indicate responses in the presence of FGF23.

**Figure 8:**
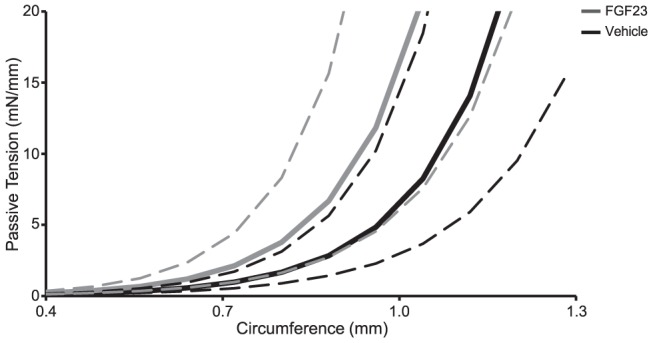
Long-term effects of FGF23 on the passive length-tension relationships. Long-term effects of FGF23 (6 ng/mL) or vehicle (DMSO 0.17% v/v) on the passive length-tension relationships. Bold solid black lines indicate responses in the presents of vehicle with thin dashed lines showing ± SEM range; bold gray lines indicate responses in the presence of FGF23 with thin dashed gray lines showing ± SEM range.

To test whether FGF23 modified vascular function, we used an *ex vivo* system where isolated arteries from WT mice were exposed to high doses of FGF23 (6 ng/mL) followed by assessment of contractile and dilatory functions during a time frame which ranged from short to long-term incubation (30 min to 3 hours) (as detailed in Material and Methods S1). Direct application of FGF23 to unstimulated mesenteric arteries had no detectable contractile effect when compared to vehicle over a period of 30 minutes (baseline tension: FGF23; 0.3±0.2 mN/mm versus vehicle; 0.0±0.3 mN/mm, p>0.05). Similarly, after contractile stimulation with 0.1 mmol/L phenylephrine (PHE), a single application of FGF23 or vehicle had no effect on contractile tension (FGF23; −0.2±0,1 mN/mm versus vehicle; −0.3±0.1 mN/mm, p>0.05) over the period of 20 minutes. Short term incubation with FGF23 or vehicle had no effect on concentration response curves induced by vasoconstrictors PHE and thromboxane receptor (TXA_2_) analog U46619 or endothelium-dependent agonist acetylcholine (ACh) and endothelium-independent agonist nitric oxide donor sodium nitroprusside (SNP) (as described in Materials and Methods S1, Figure 6). Similarly, long-term incubation with FGF23 had no effect on the concentrations-response curves to PHE; TXA_2_; ACh or SNP (Figure 7). Finally, prolonged incubation with FGF23 or vehicle had no effect on the passive length-tension relationships within a physiological range of tensions (Figure 8) suggesting no evidence in changes of structural features of vascular wall. Taken together, FGF23 did not modulate vascular functional and structural features in this model (Figure 6–8).

## Discussion

Intense research efforts have been devoted to understanding the etiology of the increased prevalence of cardiovascular morbidity and mortality in CKD. Recent data unequivocally link high circulating levels of the phosphaturic hormone FGF23 to static and dynamic assessments of vascular dysfunction and indeed FGF23 is now considered to be a predictive marker of all-cause and cardiovascular mortality in longitudinal outcome studies [2], [3], [4], [5], [6], [7], [18]. Further, circulating Klotho functions as a true hormone in that it prevents vascular calcification and protects the endothelium. These data are consistent with the observation that CKD, a Klotho-deficient state, confers increased susceptibility to vascular damage [11]. Despite a continuously growing body of clinical data linking FGF23 to cardiovascular disease, experimental data supporting direct vascular effects of FGF23 is lacking. Also, there is conflicting data whether membrane-bound Klotho is expressed locally in the vascular wall. We addressed these issues by investigating the expression of Klotho in arteries from WT mice and a novel mouse model with a targeted deletion of Klotho in VSMC, and furthermore performed functional evaluations of FGF23 treatment on vascular calcification and endothelial function *in vitro* and *ex vivo*. Collectively, we demonstrate that i) Klotho transcript and protein expression is low or absent in mouse arteries; ii) the low levels of Klotho in arteries do not mediate FGF23 signaling as evidenced by the lack of Egr-1 upregulation; iii) FGF23 does not itself influence vascular calcification or endothelium dependent and independent dilatory and contractile function, as well as structure in our experimental models.

The presence of Klotho in vascular tissue is currently a matter of debate. Donate-Correa *et al*. reported low expression level of Klotho in the thoracic aorta from patients undergoing elective cardiac surgery and Lim *et al*. detected Klotho protein in human epigastric and renal arteries and in a cell line of human vascular smooth muscle cells, whereas other preliminary reports failed to detect vascular Klotho expression [12], [19]. There are several critical factors that may account for these discrepancies, including issues relating to specificity and sensitivity of the antibodies used for Klotho detection and inconsistencies regarding the vascular segments being analyzed. Importantly, we herein demonstrate comparable low Klotho transcript level in arteries from WT, *Sm22-KL^−/−^* and *β-KL^−/−^* mice, suggesting that this represents an unspecific background signal. Additionally, using two well established anti-Klotho antibodies [20] we show that vascular Klotho protein is either absent or produced at non-detectable levels, which do not translate into a functional FGF23-Klotho signaling complex as is evidenced by the lack of Egr-1 induction after an intravenous FGF23 injection. Taken together, our data strongly support that FGF23 does not signal through a Klotho-dependent pathway in the arterial wall, at least not in mice. We acknowledge the possibility of species differences in terms of vascular Klotho expression and suggest that further extensive evaluations of human arterial specimens are warranted. However, even if assuming a weak or scattered expression of Klotho in human arteries, it appears unlikely that such expression provides the basis for a vascular FGF23 signaling that would fully explain the established association between FGF23 and vascular dysfunction.

To test the possibility that FGF23 could modulate vascular calcification or aggravate functional and structural properties independent of Klotho, we i) treated bVSMCs with FGF23 *in vitro* to investigate a potential role in the calcification process, and ii) analyzed if acute or longer term exposure to FGF23 impaired the endothelium dependent and independent dilatory and contractile responses when stimulated with pharmacological agonists or altered vascular structure as assessed by passive-length relation in maximally relaxed vessels in an *ex vivo* model. Importantly, neither of these processes were affected by FGF23 treatment, supporting that FGF23 does not modify these phenotypes independent of Klotho as previously reported in cardiomycocytes [10]. Our data suggest that FGF23 itself may not be a primary target for reducing vascular damage in CKD but rather support the concept that FGF23 portrays vascular pathology associated with a disordered mineral metabolism such as hyperphosphatemia, low levels of vitamin D and secondary hyperparathyroidism. This is also in agreement with a recent study demonstrating a pronounced increase in vascular calcification and mortality rate in a rat model of renal failure in which neutralizing FGF23 antibodies were administered [21]. Thus, based on current available data we hypothesize that FGF23 predicts cardiovascular outcomes in CKD mainly by portraying vascular stress due to parallel changes in mineral metabolism and by directly promoting left ventricular hypertrophy (LVH) [10].

In summary, arterial Klotho expression was low or absent and did not mediate vascular FGF23 signaling in mice. Furthermore, vascular calcification and function were unaffected by FGF23 treatment *in vitro* and *ex vivo* respectively, thus not supporting direct vascular effects by FGF23 as the cause of vascular pathology in CKD.

## Supporting Information

Figure S1 **Low but detectable levels of Klotho mRNA in bVSMCs.** bVSMCs were grown to confluence (day 0), whereafter cells were treated +/−5 mM BGP for up to 7 days. Low but detectable transcript levels were measured with qPCR at each time point. No differences in klotho levels were detected between +/− BGP. Data are shown as mean ± SEM.
(TIF)

Table S1 **Sequences of the primers used for genotyping and real time qPCR.**
(PDF)

Material and Methods S1 **Full description of functional studies in arteries** ***ex vivo*** **.**
(PDF)
