# Supplementary figures and images for: Arterial Klotho Expression and FGF23 Effects on Vascular Calcification and Function
Source: PLoS One. 2013 Apr 5;8(4):e60658. doi: 10.1371/journal.pone.0060658 (PMC3618102; doi:10.1371/journal.pone.0060658)

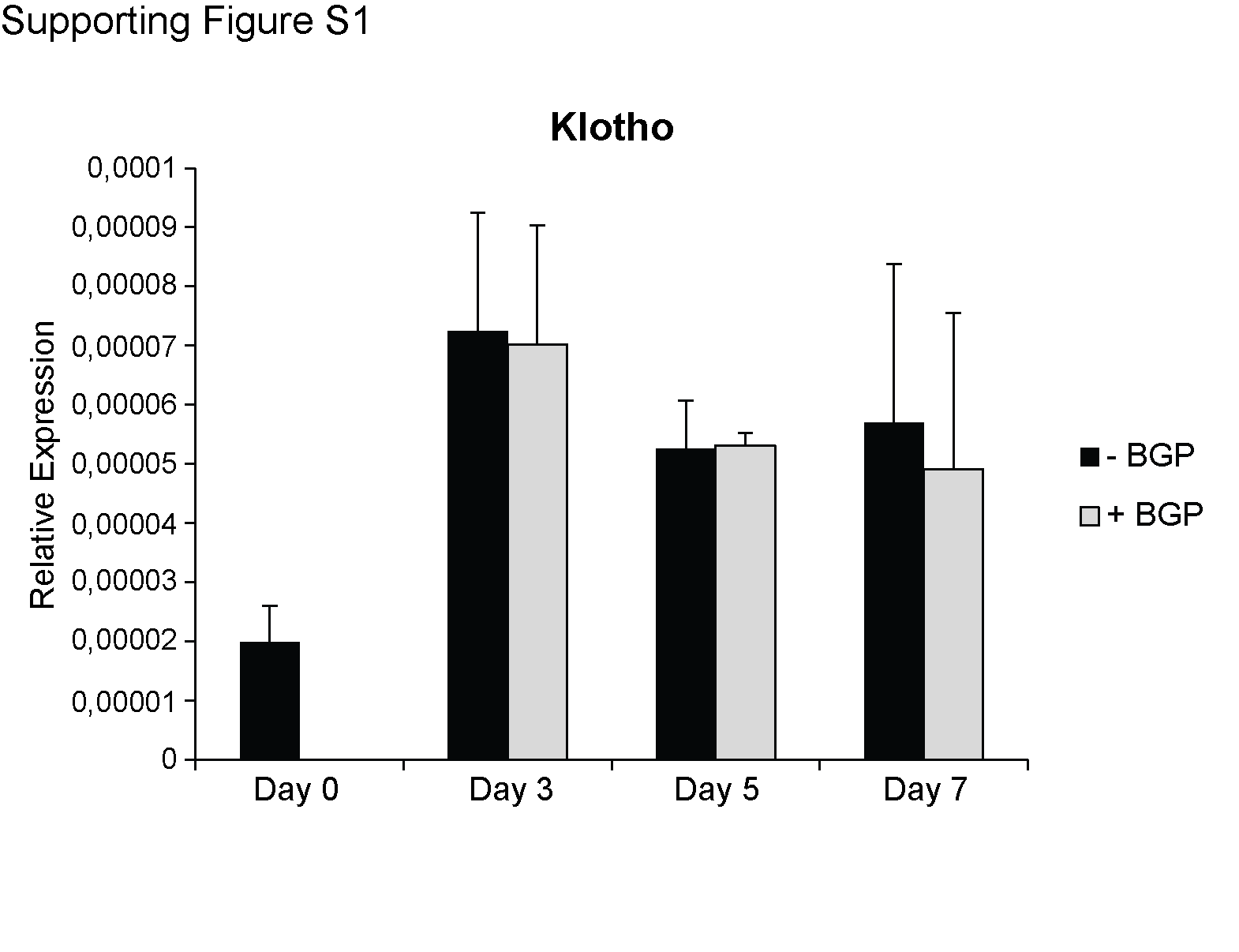

Supplement: Figure S1 — Low but detectable levels of Klotho mRNA in bVSMCs. bVSMCs were grown to confluence (day 0), whereafter cells were treated +/−5 mM BGP for up to 7 days. Low but detectable transcript levels were measured with qPCR at each time point. No differences in klotho levels were detected between +/− BGP. Data are shown as mean ± SEM. (TIF) [file pone.0060658.s001.tif]
