# Supplementary material for: Arterial Klotho Expression and FGF23 Effects on Vascular Calcification and Function
Source: PLoS One. 2013 Apr 5;8(4):e60658. doi: 10.1371/journal.pone.0060658 (PMC3618102; doi:10.1371/journal.pone.0060658)
Supplement: Table S1 — Sequences of the primers used for genotyping and real time qPCR. (PDF) [file pone.0060658.s002.pdf]

Supporting Table S1

Target gene (genotyping)	Forward	Reverse
Klotho-LoxP	TTG TCA ATA TGT AAA TAA TTT GAG CAG TAG GG	GTT GTT GAA AGA GGG AGC TAG TGG TAG TTA
Sm22-cre	CAG ACA CCG AAG CTA CTC TCC TTC C	CGC ATA ACC AGT GAA ACA GCA TTG C
**Target gene (real time qPCR)**	**Forward**	**Reverse**
B-actin	CCG TAA AGA CCT CTA TGC CAA CAC	GAG CCA CCG ATC CAC ACA GA
Klotho	TGT ATG TGA CAG CCA ATG GAA TCG	GAA TAC GCA AAG TAG CCA CAA AGG
Cre recombinase	CAG ACA CCG AAG CTA CTC TCC TTC C	CGC ATA ACC AGT GAA ACA GCA TTG C
