# Supplementary material for: Arterial Klotho Expression and FGF23 Effects on Vascular Calcification and Function
Source: PLoS One. 2013 Apr 5;8(4):e60658. doi: 10.1371/journal.pone.0060658 (PMC3618102; doi:10.1371/journal.pone.0060658)
Supplement: Material and Methods S1 — Full description of functional studies in arteries ex vivo . (PDF) [file pone.0060658.s003.pdf]

Supporting Material and Methods S1

Ex vivo testing of vascular response to FGF23

Animals and tissues

Male C57BL/6 mice aged between 8-10 weeks were euthanized by cervical dislocation. The abdomen was opened and flushed with ice-cold physiological salt solution (PSS, composition (mmol/L): NaCl, 119; KCl, 4.7; KH_2PO_4, 1.2; $NaHCO_3$, 25; $MgSO_4$, 1.2, D-glucose 5.5; Na_2EDTA, 0.03). The mesentery was carefully detached from surrounding tissues, rinsed and stored on ice in PSS. All subsequent dissection and mounting procedures were carried out in ice-cold PSS using a stereo microscope. First or 2nd order branches from the main mesenteric artery were dissected and two wires (stainless steel, diameter 0,04mm or 0.025 of tungsten) were carefully inserted into the lumen of the vessel. Vessels with wires were stored in fresh ice-cold PSS until mounting for isometric force recording in a four channel myograph (www.dmt.dk).

Isometric force recording and normalisation protocol

After mounting in 5mL wells containing cold PSS bubbled with 95%O_2/5%CO_2, the heating (37°C) was switched on and they were gently stretched until slack was taken up. The longitudinal length of the segment was then recorded (see Table 1). Segments were equilibrated at slack length for minimum 30-40 minutes, whereby the well contents were regularly replaced with fresh pre-warmed and pre-bubbled PSS.

After equilibration, an approximation of the optimal length for maximal isometric force development was determined for each individual artery segment through the use of passive length-tension relationships. The vascular segments were stretched transversally in steps which increased the internal circumference by 0.05mm or 0.1mm. Each step lasted 90s and the passive force was recorded prior the next stretch. When forces exceeded ≈15mN 90s after a step increase, the internal circumference was brought back to slack. The entire procedure took between 5 and 10 steps, depending on the vascular segment. The recorded forces were normalised to tension (mN/mm), and the relationship between the internal circumference and passive tension was fitted for each vascular segment to an exponential relationship to determine the optimal circumference (Mulvany and Halpern, 1977). Each vascular segment was then brought to its optimal internal circumference (noted as optimal diameter (opt Ø), see Table 1), and equilibrated for another 30 minutes. The passive tensions at opt Ø for the vascular segments used in this study are presented in Table 1.

All preparations were then challenged twice with high K^+-PSS (K-PSS, K^+=125mmol/L, equimolar replacement of Na^+ for 90s with 5min interval). After another 5 minutes, all segments were

preconstricted with norepinephrine (NE, 30μmol/L) and acetylcholine (ACh, 1μmol/L) was added after stabilisation of the contraction. A relaxation of >50% was taken to indicate a functionally intact endothelium. Following wash-out and stabilisation of the baseline for 5min, K-PSS was added for 5min to determine the maximal response to receptor-independent contractile activation (reference contraction). The amplitude of the NE- and K-PSS-induced contractions and the relaxations to Ach in vessel segments used in the experimental protocols are shown in Table 1.

Short-term effects of FGF23

FGF23 (6ngr/mL, 30 min) was added to one vascular segment and the vehicle (DMSO, 0.17%v/v final concentration, 30 min) to a paired control vascular segment. Concentration-response curves to PHE (1nmol/L to 0.1mmol/l) were then obtained in the presence of FGF23 or vehicle. After the final contraction to phenylephrine (PHE, 0.1mmol/L), ACh was added cumulatively (1nmol/L to 0.01mmol/L) to obtain concentration-response curves in the presence or absence of FGF23. Following wash-out, FGF23 and vehicle were re-added and concentration-response curves to thromboxane receptor TXA_2 analog U46619 (0.1nmol/L to 1μmol/L) and sodium nitroprusside (SNP, 1nmol/L to 0.1mmol/L) were obtained.

The two other vascular segments were contracted with PHE (0.1mmol/L) and single concentration of FGF23 (6ngr/mL) or vehicle **(**DMSO, 0.17%v/v final concentration**)** was added after the contraction had stabilised.

Long-term (3 hours) effects of FGF23

FGF23 (6ng/mL) or DMSO (DMSO, 0.17%v/v final concentration) were added for 180min (contents refreshed after 90min) in isolated vessel segments. When FGF23 or vehicle were finally washed out, the vessel segments were challenged twice with K-PSS after which concentration-response curves to PHE and ACh, U46619 and SNP were obtained as previously described.

Protocol for registration of passive mechanical properties

After the obtaining SNP concentration-response curves, the segments used for the long-term protocol were washed-out with Ca^{2+}-free PSS with papaverine (final concentration 0.1mmol/L) and SNP (final concentration 1mmol/L). After the passive forces had stabilised, the preparations were brought back to slack length after which a new passive length-tension relation was obtained, as described previously, in order to have indications if structural changes occur after 3 hours incubations with FGF23.

Calculations and data expression

All data are expressed as means ± SEM of N the number of independent observations. For all the data described, N=5 except where expressly mentioned otherwise. The total number of observations for any given parameter is depicted by n.

Contractions to K-PSS, U46619, NA or PHE were expressed as active force which was calculated from the baseline prior to addition of the stimulus. These data were then further normalised as % of the K-PSS reference contraction. Relaxations to ACh and SNP were calculated as % relaxation present whereby the stable level of contraction prior to addition of the vasodilators was set at 0% and the baseline obtained before the addition of the contractile stimulus as 100%. For each individual mesenteric artery segment, the data on the passive length-tension relationship, were fitted to an exponential function (Y=Aexp(bX) whereby Y is passive tension (mN/mm), A is the first fitted parameter (mN/mm), b is the second fitted parameter (mm^{-1}) and X is circumference (mm). In each group (vehicle treated and FGF23-treated) average and SEM were calculated for the A and B parameters.

Compounds and solutions

(-)-phenylephrine HCl, (-)-noradrenaline bitartrate, acetylcholine HCl, Na+-nitroprusside and U46619 were all obtained from Sigma-Aldrich Sweden (www.sigmaaldrich.se). They were stored at -15°C as stock solutions of respectively (mol/L): 0.1 in ultrapure water, 0.1 in 1mmol/L ascorbic acid; 0.1 in ultrapure water, 0.1 in ultrapure water and 0.2 in DMSO. FGF23 protein was kindly provided from Dr. Moosa Mohammadi.

References

Mulvany & Halpern (1977) Circ Res 41(1):19.

Table 1: Properties of mesenteric arteries used in this study (N=5, n=20) for each parameter;

Parameter	
Segment length (mm)	1.85±0.04
Optimal diameter (opt. Ø, mm)	0.31±0.02
Passive tension at opt. Ø. (mN/mm)	3.2±0.3
K-PSS active tension (mN/mm)	3.6±0.4
NE active tension (mN/mm)	3.5±0.4
ACh-relaxation (%)	78±5
